# Association between smoking and postoperative delirium in surgical patients with pulmonary hypertension: a secondary analysis of a cohort study

**DOI:** 10.1186/s12888-022-03981-5

**Published:** 2022-06-01

**Authors:** Sai Zhou, Shuqing Shi, Chang Xie, Gong Chen

**Affiliations:** 1 Academic Affairs Department, Changsha Medical University, Changsha, 410000 Hunan China; 2 Department of Anesthesiology, The Third Xiangya Hospital of Central South University, Changsha, 410013 Hunan China

**Keywords:** Smoking, Postoperative delirium, Pulmonary hypertension

## Abstract

**Background:**

Previous studies have declared that smoking was a risk factor for postoperative delirium (POD), but others have inconsistent results. Up till now, the association between smoking and POD has not been verified. This study investigates the relationship between smoking and POD in patients with pulmonary hypertension (PHTN) in the United States.

**Methods:**

Patients with PHTN who underwent non-cardiac, non-obstetric surgery were enrolled in the original research completed by Aalap C. et al. We further excluded the patients undergoing intracranial surgery and the patients with sepsis and perioperative stroke to avoid interference with POD assessment. The generalized linear model and generalized additive model were used to explore the relationship between smoking and POD. The propensity score adjustment was used for sensitivity analyses.

**Results:**

Five hundred thirty-nine patients were included in this study. The overall incidence of POD was 3.0% (16/539). After adjusting the potential confounders (age, systemic hypertension, coronary artery disease, COPD, length of surgery, intrathoracic surgery, vascular surgery), a positive relationship was found between smoking status and POD (OR = 4.53, 95% CI: 1.22 to 16.86, *P* = 0.0243). In addition, the curvilinear relationship between smoking burden (pack-years) and POD is close to a linear relationship.

**Conclusion:**

Smoking probably shows a positive correlation with POD in patients with PHTN.

**Supplementary Information:**

The online version contains supplementary material available at 10.1186/s12888-022-03981-5.

## Introduction

Postoperative delirium (POD) is a common neurological complication after surgery [1]. Though the incidence of POD in the general population is 2–3%, it occurs in up to 50–70% of high-risk patients [2]. POD extends the length of hospital stay by 2–3 days and increases the postoperative 30-day mortality by 7–10% [2]. Though it brings a heavy burden to individuals, families, and societies, there is a lack of effective therapeutics to treat POD. Currently, accurate identification of risk factors is the mainstay for POD prevention.

Many risk factors contribute to POD, such as old age, diabetes, COPD, and pulmonary circulation disorders [3, 4]. Tobacco smoking is a significant risk factor associated with neuropsychiatric illness including POD [5]. However, some studies reported that smoking did not significantly influence POD incidence [6–8]. Up till now, the association between smoking and POD has not been verified.

The current study conducted a secondary data analysis of a published paper to explore the association between smoking and POD [9]. The original article studied the correlation between self-reported functional status and postoperative outcomes in non-cardiac surgical patients with pulmonary hypertension (PHTN). Using this data, we aim to explore the association between tobacco smoking and POD in PHTN patients in the present study. We hypothesize that smoking patients with PHTN would have a higher risk of POD.

## Methods

### Data source

We obtain data from the "DATADRYAD" database (datadryad.org), allowing users to download raw data for free. Following the Dryad Terms of Service, we quoted the Dryad data package in this research. (Shah, Aalap C. et al. (2019), Data from Self-reported functional status predicts postoperative outcomes in non-cardiac surgery patients with pulmonary hypertension, Dryad, Dataset, https://doi.org/10.5061/dryad.9236ng5). The variables in this database file include the following: age, gender, BMI, ASA classification, self-reported functional status, PHTN severity classification, length of surgery, surgical approach, comorbidities, medications, inhalational agents, tobacco smoking, delirium and so on.

### Ethics approval and consent to participate

The participation consent and the new ethics approval were not applicable for this study since this is a secondary analysis of original research. The original authors (Aalap C. Shah et al.) had provided the ethics permission from the University of Washington.

### Study population

Aalap C. Shah et al. conducted a retrospective cohort study at the University of Washington Medical Center from April 2007 to September 2013. Patients who underwent elective non-cardiac, non-obstetric surgery were recruited in the original research. Exclusion criteria include 1) PHTN was diagnosed after the operation; 2) The patient was admitted more than 24 h before the operation; 3) The preoperative anesthesia clinic visit data including the self-reported functional status was not available; or 4) The operation was canceled before or after anesthesia. A total of 550 patients were recruited and selected in the original investigation. To avoid interference with POD assessment, we further excluded patients undergoing intracranial surgery and patients with sepsis and perioperative stroke. Finally, 539 patients were included in our study.

### Data collection

According to the description of the original paper [9], the actual data was derived from hospital records of pre-anesthesia clinics and intraoperative anesthesia. Demographic information, including age, sex, BMI, and self-reported functional status were collected. Documented comorbidities include cardiovascular disease (myocardial infarction, arrhythmia, congestive heart failure), lung disease (asthma and COPD), diabetes, and renal failure. PHTN severity was also recorded according to the WHO classification of PHTN subtypes. Some perioperative medications and factors related to surgery and anesthesia were also collected. Smoking-related records included whether they smoked or not, current smoking status, and smoking burden (pack-years).

The primary patient outcome is the occurrence of POD. The gold standard for diagnosing delirium is DSM5 (Diagnostic and Statistical Manual of Mental Disorders, 5th edition) [10]. However, the assessment approach was not documented in the raw data. POD might be diagnosed by a psychiatrist or other physician.

### Statistical analysis

Patient characteristics for different groups of smoking status and smoking burdens were reported. Continuous variables were expressed as mean (SD) or median (IQR), depending on whether the distribution was normal. Categorical variables were expressed as frequency or percentage. One-way ANOVA or Kruskal Wallis H test was used for continuous data, and the chi-square test was used for categorical data to explore statistical differences between different groups. We used never-smokers as a reference to estimate ORs with 95% confidence interval (CI) by univariate regression and multivariate regression adjusted for several confounding factors. The unadjusted, minimally adjusted, and fully adjusted analyses are all exhibited following the STROBE recommendations. The covariances adjusted in the fully adjusted model were determined by their associations with the POD (*P* < 0.1) or a change in effect estimate of more than 10% [11]. The generalized additive model (GAM) was also used to identify the curve relationship. In addition, the propensity score adjustment was used to verify the stability of multivariate analyses. All analyses were performed using software packages R (http://www.R-project.org, The R Foundation) and EmpowerStats (http://www.empowerstats.com, X&Y Solutions, Inc., Boston, MA). Two-sized *P* values less than 0.05 were considered statistically significant.

## Results

### Baseline characteristics of patients

There were 271 never-smokers and 268 ever-smokers in this cohort. Sixteen patients developed POD, 11 in the smoker group and 5 in the never-smoker group. Baseline characteristics are listed in Table 1. The age of the smoker group was slightly higher than the age of the never-smoker group. Compared with never-smokers, smokers had a significantly higher ASA classification and a higher percentage of arrhythmia and COPD. Compared with the never-smokers, more patients in the smoking group received statin, steroids, and inhalational agents. Patient characteristics of different smoking burden groups (pack-years) are listed in Table S1.

**Table 1:** Patient characteristics

Variables	Never Smoker (*n* = 271)	Smoker (*n* = 268)	*P*-value
**Age, years [mean (SD)]**	58.88 (15.07)	61.96 (12.69)	0.059
**Male gender**	140 (51.66%)	151 (56.34%)	0.275
**BMI, kg/m**^**2**^ **[mean (SD)]**	32.05 (13.43)	31.46 (10.51)	0.469
**Poor functional status (< 4 MET)**	133 (49.08%)	137 (51.12%)	0.635
**ASA classification**			0.009
** II**	28 (10.33%)	15 (5.60%)	
** III**	192 (70.85%)	177 (66.04%)	
** IV**	51 (18.82%)	76 (28.36%)	
**PHTN severity classification**			0.479
** Mild**	118 (46.09%)	108 (41.38%)	
** Moderate**	113 (44.14%)	129 (49.43%)	
** Severe**	25 (9.77%)	24 (9.20%)	
**Surgical characteristics**			
** Length of surgery [median (IQR)]**	77.00 (34.00–141.00)	94.00 (36.75–159.25)	0.168
** Open surgical approach**	153 (56.46%)	131 (48.88%)	0.078
** Intraabdominal surgery**	52 (19.19%)	60 (22.39%)	0.360
** Intrathoracic surgery**	14 (5.17%)	13 (4.85%)	0.867
** Vascular surgery**	6 (2.21%)	13 (4.85%)	0.097
**Comorbidities**			
** Systemic hypertension**	172 (63.47%)	187 (69.78%)	0.121
** Coronary artery disease**	88 (32.84%)	93 (34.83%)	0.626
** Arrhythmia**	136 (50.18%)	105 (39.18%)	0.010
** Angina**	16 (5.90%)	23 (8.58%)	0.230
** Asthma**	44 (16.24%)	31 (11.61%)	0.121
** COPD**	10 (3.70%)	61 (22.85%)	< 0.001
** Diabetes**	77 (28.41%)	78 (29.21%)	0.838
** Renal failure**	75 (27.68%)	60 (22.39%)	0.157
**Medications**			
** Anticoagulant**	82 (30.26%)	65 (24.25%)	0.118
** Antiplatelet**	9 (3.32%)	9 (3.36%)	0.981
** Statin**	110 (40.59%)	132 (49.25%)	0.043
** Steroids**	39 (14.39%)	61 (22.76%)	0.012
** Atropine**	2 (0.74%)	3 (1.13%)	0.683
** Inhalational agents**	137 (50.93%)	168 (63.40%)	0.004
** Isoflurane**	6 (2.21%)	12 (4.48%)	0.144
** Sevoflurane**	111 (40.96%)	136 (50.75%)	0.023
** Delirium**	5 (1.85%)	11 (4.10%)	0.122
** Mortality**	3 (1.11%)	4 (1.49%)	0.724

*BMI* Body mass index, *MET* Metabolic equivalent of task, *ASA* American Society of Anesthesiologists, *PHTN* Pulmonary hypertension, *IQR* Interquartile range, *COPD* Chronic obstructive pulmonary disease

### The relationship between the smoking status and POD

The univariate analysis showed that only age and intrathoracic surgery were correlated with POD (Table S2). To accurately evaluate the association between smoking status and POD, we exhibited the unadjusted and adjusted models in Table 2. In the unadjusted model, tobacco smoking did not significantly correlate with POD (OR = 2.28, 95% CI: 0.78 to 6.64, *P* = 0.1320). In the minimally adjusted model, the association between smoking and POD was approached but not significant (OR = 1.76, 95% CI: 0.58 to 5.29, *P* = 0.3164). However, smoking showed a positive correlation with POD in the fully adjusted model (OR = 4.53, 95% CI: 1.22 to 16.86, *P* = 0.0243).

**Table 2:** Relationship between smoking status and POD in different models

Variables	Unadjusted model (OR 95%CI *P*)	Minimally adjusted model (OR 95%CI *P*)	Fully adjusted model (OR 95%CI *P*)
**Tobacco Smoking**			
**Never smoker**	ref	ref	ref
** Smoker**	2.28 (0.78, 6.64) 0.1320	1.76 (0.58, 5.29) 0.3164	4.53 (1.22, 16.86) 0.0243

Unadjusted model: We do not adjust for other covariances

Minimally adjusted model: We adjust for age and length of surgery

Fully adjusted model: We adjust for age, length of surgery, intrathoracic surgery, vascular surgery, COPD, systemic hypertension, and coronary artery disease

### The relationship between the smoking burden and POD

**Fig. 1:**
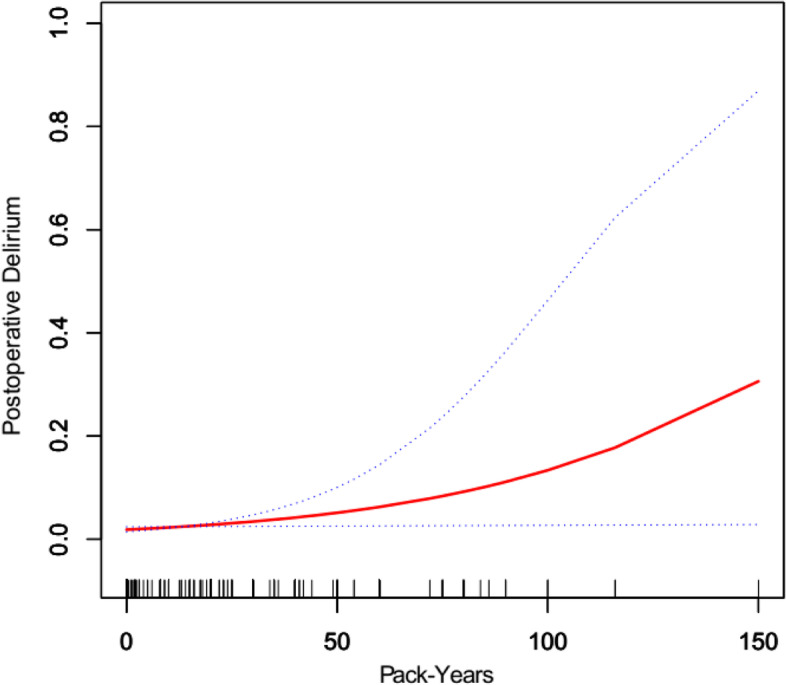
The relationship between smoking burden and postoperative delirium. A curvilinear relationship between smoking burden and POD is close to a linear relationship after adjusting for age, length of surgery, intrathoracic surgery, COPD, coronary artery disease, systemic hypertension, and vascular surgery

Next, we compared the incidence of POD in different smoking burdens. Since the smoking burden was continuous data, we explored the curve relationship between pack-years and POD. After adjusting for several confounders (age, length of surgery, COPD, coronary artery disease, systemic hypertension, vascular surgery, and intrathoracic surgery), we found a curvilinear relationship between smoking burden and POD was close to a linear relationship (Fig. 1).

### Sensitivity analyses

To verify the stability of our results, we further conducted propensity score-adjusted regression for sensitivity analyses. POD and non-POD patients were matched 1:20 by propensity score, which yielded a cohort of 6 patients in the POD group and 120 in the non-POD group. The patient characteristics of the two groups after propensity score matching are listed in Table S3. After adjusting for the propensity score, the correlation between tobacco smoking and POD approached significant (OR = 7.36, 95% CI: 0.75 to 72.47, *P* = 0.0870). The results of propensity score-adjusted analyses are listed in Table S4.

## Discussion

This study explored the relationship between smoking and POD in PHTN patients. The fully adjusted model shows that smoking may be positively correlated with POD. In addition, we also found the curvilinear relationship between pack-years of smoking and POD.

We performed a PubMed search using both the keywords "smoking" and "postoperative delirium." As of October 2021, 52 scientific papers were retrieved from this database, but only eight were related to our research. In those studies, smoking was listed as a risk factor for POD in patients with different types of surgery. However, they did not sufficiently adjust the potential confounders for the association between smoking and POD. These confounders included COPD, coronary artery disease, hypertension and so on. Therefore, their conclusions were limited because the confounding factors above were closely related to smoking and POD. The present study is the first to verify the association between smoking and POD to the best of our knowledge.

The incidence of POD in the present study was 3.0%, which was lower than recently reported in the literature [12]. This discrepancy may be due primarily to different study populations. Most POD studies focused on the elderly population, whose frequency was significantly higher than the younger adults [12]. However, age was only required to be an adult in this study. The incidence reported in the literature was 2–3% in the general population, close to our reported incidence [2]. In addition, the method for POD assessment was not provided in sufficient detail in the original paper [9], which may have contributed to differences in the rate of POD findings.

Our results suggested a probable correlation between tobacco smoking and POD. A previous study has shown that smoking leads to several mental disorders such as cognitive dysfunction [13]. In the present study, after adjusting for several covariates, the risk of POD was 3.53 times higher in smokers than in never-smokers. However, due to many adjusted variables, the confidence interval of the adjusted odds ratio was broad. To verify the stability of the results, we used propensity adjustment for sensitivity analyses, which exhibited steady odds ratios and near-significant correlation. In addition, the curve relationship between smoking burden and POD showed that the higher number of pack-years smoked, the greater risk of developing POD. Therefore, individuals with a heavy smoking burden might require special attention for POD prevention. It was reported that some agents such as dexmedetomidine might be effective [14].

Our research has several advantages. First, the generalized linear model and the generalized additive model were used to clarify the relationships between smoking and POD, which will help us better discover the actual connection between exposure and results. Second, we used strict statistical adjustments to minimize residual confounding. Although previous studies have suggested that smoking was a risk factor for POD, they have not adjusted the necessary confounding factors. We selected 26 possible confounding factors from the original data table and screened the covariates according to the methods provided in the previous literature [11] to determine the precisely adjusted confounding. Third, in the selection of covariates, continuous variables and categorical variables were used to verify the association between smoking and POD.

Our research has some limitations. First, due to the limit of the raw data, we cannot determine the diagnosing method of POD. The POD might be evaluated by a psychiatrist or other doctor. Second, because the study population only included Americans with PHTN, it may not be generalized to people from different countries and patients with other diseases. Third, this study did not include many drugs that affect POD, such as midazolam, dexmedetomidine, and polypharmacy [14–16], owing to the limitation of the original data. Fourth, due to the low incidence and number of cases in our study, only 6 POD patients and 120 non-POD patients were obtained after propensity score matching. Thus, the correlation was only approached but not significant in the propensity score-adjusted regression analyses. However, the OR value remained at 7.36, indicating a stable trend in the association between smoking and POD. Therefore, smoking probably shows a positive correlation with POD in patients with PHTN. The question remains open for further studies.

## Conclusion

Smoking probably shows a positive correlation with POD in patients with PHTN.

## Supplementary Information

**Additional file 1.**

**Additional file 2.**

**Additional file 3.**

**Additional file 4.**

## Data Availability

The data used to support the findings of this study are available from the DATADRYAD website.
