# Supplementary material for: Association between smoking and postoperative delirium in surgical patients with pulmonary hypertension: a secondary analysis of a cohort study
Source: BMC Psychiatry. 2022 Jun 1;22:371. doi: 10.1186/s12888-022-03981-5 (PMC9158079; doi:10.1186/s12888-022-03981-5)
Supplement: Supplementary file 1 — Additional file 1. [file 12888_2022_3981_MOESM1_ESM.docx]

**Table S1: Basic characteristics of different smoking burden groups**

| Variables | Pack-years=0  (n=271) | Pack-years>0, <=50  (n=157) | Pack-years>50, <=100  (n=34) | Pack-years>100  (n=3) | *P*-value |
| --- | --- | --- | --- | --- | --- |
| Age, years [mean (SD)] | 58.88 (15.07) | 61.69 (12.58) | 63.91 (10.70) | 72.33 (2.31) | 0.062 |
| Male gender | 140 (51.66%) | 97 (61.78%) | 17 (50.00%) | 1 (33.33%) | 0.169 |
| BMI, kg/m2 [mean (SD)] | 32.05 (13.43) | 30.81 (10.78) | 32.91 (10.46) | 26.36 (2.93) | 0.108 |
| Poor functional status (<4 MET) | 133 (49.08%) | 68 (43.31%) | 25 (73.53%) | 1 (33.33%) | 0.015 |
| ASA classification |  |  |  |  | <0.001 |
| II | 28 (10.33%) | 7 (4.46%) | 1 (2.94%) | 0 (0.00%) |  |
| III | 192 (70.85%) | 111 (70.70%) | 16 (47.06%) | 0 (0.00%) |  |
| IV | 51 (18.82%) | 39 (24.84%) | 17 (50.00%) | 3 (100.00%) |  |
| PHTN severity classification |  |  |  |  | 0.034 |
| Mild | 118 (46.09%) | 75 (49.67%) | 8 (23.53%) | 0 (0.00%) |  |
| Moderate | 113 (44.14%) | 63 (41.72%) | 24 (70.59%) | 3 (100.00%) |  |
| Severe | 25 (9.77%) | 13 (8.61%) | 2 (5.88%) | 0 (0.00%) |  |
| Surgical characteristics |  |  |  |  |  |
| Length of surgery [median (IQR)] | 77.00 (34.00-141.00) | 90.00 (37.00-159.00) | 103.00 (24.25-158.00) | 185.00 (171.50-197.50) | 0.192 |
| Open surgical approach | 153 (56.46%) | 83 (52.87%) | 15 (44.12%) | 2 (66.67%) | 0.524 |
| Intraabdominal surgery | 52 (19.19%) | 29 (18.47%) | 12 (35.29%) | 0 (0.00%) | 0.109 |
| Intrathoracic surgery | 14 (5.17%) | 8 (5.10%) | 2 (5.88%) | 1 (33.33%) | 0.197 |
| Vascular surgery | 6 (2.21%) | 7 (4.46%) | 1 (2.94%) | 0 (0.00%) | 0.613 |
| Comorbidities |  |  |  |  |  |
| Systemic hypertension | 172 (63.47%) | 115 (73.25%) | 21 (61.76%) | 3 (100.00%) | 0.103 |
| Coronary artery disease | 88 (32.84%) | 58 (37.18%) | 11 (32.35%) | 1 (33.33%) | 0.828 |
| Arrhythmia | 136 (50.18%) | 63 (40.13%) | 10 (29.41%) | 1 (33.33%) | 0.047 |
| Angina | 16 (5.90%) | 12 (7.64%) | 5 (14.71%) | 1 (33.33%) | 0.087 |
| Asthma | 44 (16.24%) | 17 (10.90%) | 1 (2.94%) | 0 (0.00%) | 0.094 |
| COPD | 10 (3.70%) | 35 (22.44%) | 15 (44.12%) | 1 (33.33%) | <0.001 |
| Diabetes | 77 (28.41%) | 37 (23.57%) | 16 (47.06%) | 0 (0.00%) | 0.031 |
| Renal failure | 75 (27.68%) | 28 (17.83%) | 9 (26.47%) | 0 (0.00%) | 0.097 |
| Medications |  |  |  |  |  |
| Anticoagulant | 82 (30.26%) | 38 (24.20%) | 6 (17.65%) | 0 (0.00%) | 0.196 |
| Antiplatelet | 9 (3.32%) | 6 (3.82%) | 2 (5.88%) | 0 (0.00%) | 0.875 |
| Statin | 110 (40.59%) | 78 (49.68%) | 19 (55.88%) | 2 (66.67%) | 0.126 |
| Steroids | 39 (14.39%) | 35 (22.29%) | 9 (26.47%) | 1 (33.33%) | 0.091 |
| Atropine | 2 (0.74%) | 1 (0.64%) | 1 (2.94%) | 0 (0.00%) | 0.598 |
| Inhalational agents | 137 (50.93%) | 99 (63.46%) | 22 (68.75%) | 3 (100.00%) | 0.013 |
| Isoflurane | 6 (2.21%) | 8 (5.10%) | 2 (5.88%) | 0 (0.00%) | 0.356 |
| Sevoflurane | 111 (40.96%) | 82 (52.23%) | 17 (50.00%) | 3 (100.00%) | 0.030 |
| Delirium | 5 (1.85%) | 5 (3.18%) | 2 (5.88%) | 1 (33.33%) | 0.006 |
| Mortality | 3 (1.11%) | 2 (1.27%) | 1 (2.94%) | 0 (0.00%) | 0.840 |
