# Supplementary material for: Association between smoking and postoperative delirium in surgical patients with pulmonary hypertension: a secondary analysis of a cohort study
Source: BMC Psychiatry. 2022 Jun 1;22:371. doi: 10.1186/s12888-022-03981-5 (PMC9158079; doi:10.1186/s12888-022-03981-5)
Supplement: Supplementary file 2 — Additional file 2. [file 12888_2022_3981_MOESM2_ESM.docx]

**Table S2: The results of univariate analysis**

|  | Statistics | Effect size (OR) | *P*-value |
| --- | --- | --- | --- |
| Age | 60.41 ± 14.01 | 1.06 (1.01, 1.11) | 0.0128 |
| Sex |  |  |  |
| Male | 291 (53.99%) | ref |  |
| Female | 248 (46.01%) | 0.52 (0.18, 1.53) | 0.2366 |
| Tobacco smoking |  |  |  |
| Never-smokers | 271 (50.28%) | ref |  |
| Smokers | 268 (49.72%) | 2.28 (0.78, 6.64) | 0.1320 |
| BMI | 31.75 ± 12.06 | 0.98 (0.93, 1.04) | 0.4832 |
| Poor functional status |  |  |  |
| > 4 MET | 269 (49.91%) | ref |  |
| < 4 MET | 270 (50.09%) | 2.24 (0.77, 6.54) | 0.1394 |
| PHTN severity classification |  |  |  |
| Mild | 226 (43.71%) | ref |  |
| Moderate | 242 (46.81%) | 1.71 (0.56, 5.17) | 0.3443 |
| Severe | 49 (9.48%) | 1.88 (0.35, 9.99) | 0.4584 |
| Length of surgery | 113.82 ± 109.99 | 1.00 (1.00, 1.01) | 0.0581 |
| Open surgical approach |  |  |  |
| No | 255 (47.31%) | ref |  |
| Yes | 284 (52.69%) | 1.51 (0.54, 4.23) | 0.4280 |
| Intraabdominal surgery |  |  |  |
| No | 427 (79.22%) | ref |  |
| Yes | 112 (20.78%) | 0.54 (0.12, 2.39) | 0.4145 |
| Intrathoracic surgery |  |  |  |
| No | 512 (94.99%) | ref |  |
| Yes | 27 (5.01%) | 7.25 (2.17, 24.21) | 0.0013 |
| Vascular surgery |  |  |  |
| No | 520 (96.47%) | ref |  |
| Yes | 19 (3.53%) | 0.00 (0.00, Inf) | 0.9919 |
| Systemic hypertension |  |  |  |
| No | 180 (33.40%) | ref |  |
| Yes | 359 (66.60%) | 0.64 (0.23, 1.74) | 0.3764 |
| Coronary artery disease |  |  |  |
| No | 354 (66.17%) | ref |  |
| Yes | 181 (33.83%) | 0.48 (0.13, 1.72) | 0.2607 |
| Arrhythmia |  |  |  |
| No | 298 (55.29%) | 1.0 |  |
| Yes | 241 (44.71%) | 1.61 (0.59, 4.40) | 0.3502 |
| Angina |  |  |  |
| No | 500 (92.76%) | ref |  |
| Yes | 39 (7.24%) | 0.85 (0.11, 6.62) | 0.8774 |
| Asthma |  |  |  |
| No | 463 (86.06%) | ref |  |
| Yes | 75 (13.94%) | 0.00 (0.00, Inf) | 0.9896 |
| COPD |  |  |  |
| No | 466 (86.78%) | ref |  |
| Yes | 71 (13.22%) | 0.00 (0.00, Inf) | 0.9899 |
| Diabetes |  |  |  |
| No | 383 (71.19%) | ref |  |
| Yes | 155 (28.81%) | 1.13 (0.39, 3.30) | 0.8269 |
| Renal failure |  |  |  |
| No | 404 (74.95%) | ref |  |
| Yes | 135 (25.05%) | 1.37 (0.47, 4.03) | 0.5625 |
| Anticoagulant |  |  |  |
| No | 392 (72.73%) | ref |  |
| Yes | 147 (27.27%) | 1.63 (0.58, 4.56) | 0.3554 |
| Antiplatelet |  |  |  |
| No | 521 (96.66%) | ref |  |
| Yes | 18 (3.34%) | 0.00 (0.00, Inf) | 0.9922 |
| Statin |  |  |  |
| No | 297 (55.10%) | ref |  |
| Yes | 242 (44.90%) | 1.60 (0.59, 4.36) | 0.3581 |
| Steroids |  |  |  |
| No | 439 (81.45%) | ref |  |
| Yes | 100 (18.55%) | 0.62 (0.14, 2.77) | 0.5309 |
| Atropine |  |  |  |
| No | 530 (99.07%) | ref |  |
| Yes | 5 (0.93%) | 0.00 (0.00, Inf) | 0.9903 |
| Anesthesia inhalational agents |  |  |  |
| No | 229 (42.88%) | ref |  |
| Yes | 305 (57.12%) | 2.30 (0.73, 7.24) | 0.1530 |
| Isoflurane |  |  |  |
| No | 521 (96.66%) | ref |  |
| Yes | 18 (3.34%) | 1.98 (0.25, 15.90) | 0.5187 |
| Sevoflurane |  |  |  |
| No | 292 (54.17%) | ref |  |
| Yes | 247 (45.83%) | 1.19 (0.44, 3.21) | 0.7340 |
