# Supplementary material for: Association between smoking and postoperative delirium in surgical patients with pulmonary hypertension: a secondary analysis of a cohort study
Source: BMC Psychiatry. 2022 Jun 1;22:371. doi: 10.1186/s12888-022-03981-5 (PMC9158079; doi:10.1186/s12888-022-03981-5)
Supplement: Supplementary file 3 — Additional file 3. [file 12888_2022_3981_MOESM3_ESM.docx]

**Table S3: Patient characteristics after propensity score matching on POD occurrence**

| Variables | Non-POD  (n=120) | POD  (n=6) | *P* value |
| --- | --- | --- | --- |
| Age, years (mean±SD) | 60.17 ± 11.42 | 66.50 ± 10.35 | 0.1858 |
| Male gender (%) | 59 (49.2) | 3 (50) | 1.0000 |
| BMI, kg/m^2^ (mean±SD) | 33.58 ± 16.81 | 29.85 ± 4.68 | 0.5891 |
| Poor functional status, <4 MET (%) | 61 (50.8) | 2 (33.3) | 0.6757 |
| Tobacco smoking (%) | 63 (52.5) | 5 (83.3) | 0.2895 |
| PHTN severity classification (%) |  |  | 0.2905 |
| Mild | 44 (36.7) | 4 (66.7) |  |
| Moderate | 60 (50) | 2 (33.3) |  |
| Severe | 16 (13.3) | 0 (0) |  |
| Surgical characteristics |  |  |  |
| Length of surgery (mean±SD) | 115.13 ± 111.25 | 174.83 ± 184.29 | 0.2173 |
| Open surgical approach (%) | 62 (51.7) | 3 (50) | 1.0000 |
| Intraabdominal surgery (%) | 24 (20) | 2 (33.3) | 0.7866 |
| Intrathoracic surgery (%) | 0 (0) | 0 (0) | NA |
| Vascular surgery (%) | 7 (5.8) | 0 (0) | 1.0000 |
| Comorbidities |  |  |  |
| Systemic hypertension (%) | 81 (67.5) | 5 (83.3) | 0.7160 |
| Coronary artery disease (%) | 52 (43.3) | 0 (0) | 0.0931 |
| Arrhythmia (%) | 51 (42.5) | 3 (50) | 1.0000 |
| Angina (%) | 5 (4.2) | 1 (16.7) | 0.6738 |
| Asthma (%) | 31 (25.8) | 0 (0) | 0.3430 |
| COPD (%) | 31 (25.8) | 0 (0) | 0.3430 |
| Diabetes (%) | 40 (33.3) | 2 (33.3) | 1.0000 |
| Renal failure (%) | 31 (25.8) | 0 (0) | 0.3430 |
| Medications |  |  |  |
| Anticoagulant (%) | 31 (25.8) | 2 (33.3) | 1.0000 |
| Antiplatelet (%) | 8 (6.7) | 0 (0) | 1.0000 |
| Statin (%) | 49 (40.8) | 3 (50) | 0.9839 |
| Steroids (%) | 23 (19.2) | 2 (33.3) | 0.7454 |
| Atropine (%) | 4 (3.3) | 0 (0) | 1.0000 |
| Inhalational agents (%) | 65 (54.2) | 4 (66.7) | 0.8571 |
| Isoflurane (%) | 4 (3.3) | 1 (16.7) | 0.5746 |
| Sevoflurane (%) | 54 (45) | 2 (33.3) | 0.8884 |
