# Supplementary material for: Association between smoking and postoperative delirium in surgical patients with pulmonary hypertension: a secondary analysis of a cohort study
Source: BMC Psychiatry. 2022 Jun 1;22:371. doi: 10.1186/s12888-022-03981-5 (PMC9158079; doi:10.1186/s12888-022-03981-5)
Supplement: Supplementary file 4 — Additional file 4. [file 12888_2022_3981_MOESM4_ESM.docx]

**Table S4: Logistic regression model adjusted for propensity score on POD**

| Variables | Non-adjusted model (OR 95%CI *P*) | Adjust model (OR 95%CI *P*) |
| --- | --- | --- |
| Smoking status |  |  |
| Never-smoker | ref | ref |
| Smoker | 4.52 (0.51, 39.88) 0.1741 | 7.36 (0.75, 72.47) 0.0870 |

Non-adjusted model adjust for: None
Adjust model adjust for: Propensity score on POD
